# Synergistic Effects of Nano-ZnO and Low pH of Sea Water on the Physiological Energetics of the Thick Shell Mussel *Mytilus coruscus*

**DOI:** 10.3389/fphys.2018.00757

**Published:** 2018-06-19

**Authors:** Yueyong Shang, Yawen Lan, Zekang Liu, Hui Kong, Xizhi Huang, Fangli Wu, Liping Liu, Menghong Hu, Wei Huang, Youji Wang

**Affiliations:** ^1^National Demonstration Center for Experimental Fisheries Science Education, Shanghai Ocean University, Shanghai, China; ^2^Key Laboratory of Exploration and Utilization of Aquatic Genetic Resources, Ministry of Education, Shanghai Ocean University, Shanghai, China; ^3^International Research Center for Marine Biosciences at Shanghai Ocean University, Ministry of Science and Technology, Shanghai, China; ^4^Key Laboratory of Marine Ecosystem and Biogeochemistry, Second Institute of Oceanography, State Oceanic Administration, Hangzhou, China; ^5^State Key Laboratory of Satellite Ocean Environment Dynamics, Second Institute of Oceanography, State Oceanic Administration, Hangzhou, China

**Keywords:** Nano-ZnO, acidification, *Mytilus coruscus*, physiology, combined effects

## Abstract

In order to investigate the ecotoxicological effects of nano-ZnO particles and seawater acidification on marine bivalves, the thick shell mussels, *Mytilus coruscus* were subjected to joint treatments with different nano-ZnO concentrations (0 [control], 2.5 [medium] and 10 mg L^-1^ [high]) under two pH levels (7.7 [low]and 8.1 [control]) for 14 days. The results showed that respiration rate (RR), absorption efficiency (AE), clearance rate (CR), O:N ratio and scope for growth (SFG) were significantly reduced with nano-ZnO concentration increase, but ammonium excretion rate (ER) was increased. Low pH significantly reduced CR, RR, SFG, and O:N ratio of the mussels especially under high nano-ZnO conditions, and significantly increased ER. Principal component analysis (PCA) showed consistent relationships among most tested parameters, especially among SFG, RR, O:N ratio and CR under the normal pH and 0 nano-ZnO conditions. Therefore, seawater acidification and nano-ZnO interactively impact the ecophysiological responses of mussels and cause more severe effects when they appear concurrently.

## Introduction

Since the beginning of industrial revolution, large amounts of fossil fuels have been burned and the green vegetation has been reduced greatly worldwide, resulting in dramatic increased carbon dioxide (CO_2_) emissions (Caldeira and Wickett, 2003; Intergovernmental Panel on Climate Change [IPCC], 2014). The ocean plays an important role in the global carbon cycle, and about one-third of the CO_2_ emitted by human activities is absorbed by the oceans (Feely et al., 2004). The absorption of CO_2_ in the ocean slows down the trend of rising atmospheric carbon dioxide concentration, but the continuous absorption of CO_2_ changes the carbon dioxide-carbonate system of seawater, resulting in increased concentrations of hydrogen ions, CO_2_ and bicarbonate in seawater, and decreased carbonate concentration, ultimately ocean acidification (OA) (Sabine et al., 2004; Wang et al., 2012). According to the current energy use structure, it is estimated that by 2100, the ocean’s pH will be further reduced by 0.3–0.4 units, and by 0.7 units to 2300 years (Orr et al., 2005). According to the exiting evidences, OA can affect the physiological process and fitness of marine animals through acid-base regulation, digestion, metabolism and growth (Fabry et al., 2008; Melzner et al., 2009; Kroeker et al., 2010; Lannig et al., 2010; Fernández-Reiriz et al., 2011; Gazeau et al., 2013; Matoo et al., 2013; Stumpp et al., 2013; Jakubowska and Normant, 2015; Hu et al., 2015; Jansson et al., 2015).

Most nanoparticle contaminants can be deposited in water, posing a threat to aquatic ecosystem (Renzi and Guerranti, 2015). Due to the widespread use of zinc oxide nanoparticles, coastal waters and the ocean would become an ultimate sink of these environmental contaminants (Yung et al., 2014). The application of nanoparticles has attracted more and more attention in aquatic environmental research (Montes et al., 2012; Gagné et al., 2016). It has been found that nanoparticles exert immunotoxic effects on shellfish cells, such as altering DNA structure, transcription level of immune-related genes and expression of related proteins in shellfish, and inhibiting development of shellfish embryos and larvae (Wang et al., 2014). Particularly, nano-ZnO stimulates the respiration rate (RR) and reduces the time of survival of mussels (Hanna et al., 2013). Also, Muller et al. (2014) pointed out that long-term exposure of *Mytilus galloprovincialis* to nano-ZnO cause accumulation of zinc in mussel tissues, affecting the energy budget. Although nano-ZnO has attracted some attention in ecotoxicological research, there is little research on the combined physiological effects of nano-ZnO associated with additional environmental stressors on marine bivalves. Therefore, it is necessary to explore the combined effects of nano-ZnO and OA on marine species.

*Mytilus coruscus* is an important marine bivalve species in the East China Sea. The individual is large with poor mobility and long life history, and can accumulate environmental pollutants (such as heavy metals), with strong tolerance to environmental disturbance, thus often serves as an environmental indicator to monitor the changes of coastal environment (Foy et al., 2001; Reeburgh, 2007; Tian et al., 2011; Liu et al., 2014). Scope for growth (SFG), referring to the net energy balance of molluscs, is defined as the energy transferred from the diet into growth by deducting the energy consumed by respiration and excretion, which can be determined by integrating some physiological parameters (i.e., clearance, absorption, respiration, and excretion, Bayne and Widdows, 1978; Widdows and Hawkins, 1989; Smaal and Widdows, 1994; Widdows et al., 2002; Widdows and Staff, 2006; Sarà et al., 2008; Halldórsson et al., 2008; Fernández-Reiriz et al., 2011). These physiological indicators, are not only able to predict the growth rate very well (Bayne et al., 1979), but also good indicators of the health state and the sensitive indexes of the mussels to the environmental changes (Wang et al., 2012). The purpose of this study was to probe the interactive effects of decreased pH and nano-ZnO on the physiological energetic in the mussel *M. coruscus*. The physiological parameters of the shellfish were determined, including clearance rate (CR), RR, absorption efficiency (AE), ammonium excretion rate (ER), SFG, and O:N ratio.

## Materials and Methods

### Preparation of Nano-ZnO

Nano-ZnO powder (declared purity of 99.9%) was purchased in Horsehead Company, United States. It was made into stock solution for preservation (10 g nano-ZnO L^-1^). The morphology and particle size of nano-ZnO were measured by transmission electron microscope (Low Voltage Tem5, LVEM5) and scanning electron microscopy (Hitachi JSM-7500F). X-ray diffraction of nano-ZnO was determined by X’Pert PRO X-ray Diffractometer (PANalytical B.V.). Particle size range and zeta potential of nano-ZnO were determined by dynamic light scattering (DLS) using a ZetaSizer Nano ZEN3600 (Malvern, United Kingdom).

### Experimental Mussels and Acclimation Procedure

Adult mussels (shell length: 8.0 ± 2.0 cm; dry weight 1.6 ± 0.9 g) were collected from the Gouqi island, Zhejiang Province, China, acclimated for 1 week in full aeration seawater (temperature: 20 ± 0.5°C; pH: 8.1; salinity: 25 ± 1 psu), and fed daily with the microalgae *Chlorella* spp. (2.5 × 10^5^cells mL^-1^). The handling of experimental mussels was carried on in terms of regulations of the animal welfare for scientific research made by the Institutional Animal Care and Use Committee (IACUC) of Shanghai Ocean University.

### Experimental Design

The mussels were placed in six treatments: two values of pH [7.7 as the low value of present natural variability at the sampling site (Li et al., 2014), 8.1 as the present average pH] and two doses (mg L^-1^) of nano-ZnO (0 as control, 2.5 as medium, and 10.0 as high). The concentrations of 1–10 mg L^-1^ n-ZnO are the most common concentrations currently used in laboratory research, which allow for sub-lethal physiological effects over the exposure period rather than animal mortality (Ciacci et al., 2012; Hao and Chen, 2012). The mussels were divided into six treatments randomly, the six treatments were carried out in exactly the same circulatory system, each system set up three repeated experimental tanks, and each tank contained 30 mussels, with the above mentioned microalgae feed. The low pH value was reduced by adding pure CO_2_ through a pCO_2_/pH system (DAQ-M), which was equipped with pH meter (WTW 3310) and pH electrodes (SenTix 41) and operated by CapCTRL software (Loligo Systems Inc.). A multiparameter instrument (model 5200A, YSI, United States) was used to measure seawater salinity. Total alkalinity (TA) was measured by titration method. Other carbonate chemical parameters of seawater (dissolved inorganic carbon (DIC), pCO_2_, calcite saturation state (Ωcal) and aragonite saturation state (Ωara)) were calculated based on TA and pH_NBS_ using CO_2_SYS (Lewis et al., 2013).

### Physiological Measurements

#### Clearance Rate

The thick shell mussels were fasted for 6 h to clear the intestine. They were acclimated for 15 min to ensure they open shells and exchange seawater with the outside environment. Then the microalgae with the initial concentration of 2.5 × 10^4^ cells mL^-1^ were added, and no pseudo-feces were produced in a preliminary experiment. Three same experimental tanks without mussels were used as the control. At the beginning of the experiment, an initial 20 ml of seawater was taken from the each experimental tank using pipets, after mussels were fed for 60 min, 20 ml of water sample was taken from each experimental tank. The cell concentration in seawater sample was measured with a Coulter Counter (Multisizer 3, Beckman, Irvine, CA, United States). There was no significant decrease in cell concentration during the experiment in the control tanks. CR was computed by the following formula of (Coughlan, 1969):

$$\text{CR=}V\times (\text{ln}C_{\text{0}}\text{-lnCt})\text{/Nt}$$

where CR denotes the CR (L h^-1^ g^-1^), *V* represents the volume of seawater in the tanks (*L*), *C*_0_ is the initial cell concentration (cells mL^-1^), Ct is the cell concentration at time *t* (cells mL^-1^), N is the number of animals in the tank, *t* is the sampling time (*h*). CR and other subsequent physiological parameters were standardized to unit dry weight.

#### Absorption Efficiency

After measuring CR, the feces of each replicate tank were collected to calculate AE. Four liters of seawater containing 2.5 × 10^4^ cell mL^-1^ algae were filtered by 40 mm glass fiber filters (Whatman GF/C), which was ashed and pre-weighed, to determined the organic content of microalgae. The filter papers were rinsed with ammonium formate solution (0.5 M) and dried in the oven (110°C) for 24 h, the first weighing results were recorded. Another weight was measured after ashing the filter papers in a muffle furnace (450°C) for 6 h. The filters were cooled in desiccators in advance. Feces were collected by a pipet from the experimental tanks 8 h after the CR determinations, and the organic content of the feces was measured using the same method as above. AE was computed according to Conover (1966):

AE=(F-E)/[(1-E)×F]

Where AE denotes the AE (%), F represents the ratio of organic dry weight: dry weight in the diets, and E is the ratio of organic dry weight: dry weight in the feces.

Ingestion rate (IR) was computed by multiplying CR by POM concentration (particulate organic matter, mg L^-1^, Hawkins et al., 1998), i.e., the amount of ingested organic food per hour. The POM concentration was transformed to joules using a conversion value of 23 J mg^-1^ for *Chlorella spp* (Widdows and Johnson, 1988; Widdows et al., 1990).

#### Respiration Rate

The RR of mussels was measured from the corresponding treatment tanks in closed glass respirometer (800 ml) containing air-saturated seawater. To make sure that every mussel was breathing normally, the experiment began after their valves had been open for 15 min and then sealed off the respirometers for 60 min. Two tanks filled only with seawater were used as the control. The decline in oxygen content was measured by an oxygen meter (model 5200A, YSI, United States). Then the initial and final oxygen concentrations of each tank were obtained.

The RR was then computed using the following equation (Wang et al., 2015):

$$\text{RR}\;\text{=}\;[{\text{C}}_{\text{t0}}\text{-}\;C_{\text{t1}}]\;\times \;V/\text{Nt}$$

Where RR demotes the RR (mg O_2_ h^-1^d final DO levels (mg O_2_ L^-1^), respectively, *V* (*L*) is the water volume in the respirometer, *N* is the mussel number, and *t* (*h*) is the exposure time. Values for RR were changed to J h^-1^ using a conversion value of 13.98 J mg O_2_^-1^ (Wong and Cheung, 2003).

#### Ammonia Excretion Rate

Excretion rates (ER) were measured after measuring RR of the same mussels. Water samples from each tank were frozen to -20°C until the analysis. The concentration of ammonia was measured by spectrophotometry, referring to the method of phenol-sodium hypochlorite (Solorzano, 1969). ER was calculated on the basis of the difference in the concentration of ammonia in the experimental tank and the blank tank using the following equation (Wang et al., 2015):

$$\text{ER=}(C_{\text{e}}\text{-}C_{\text{c}})\times (V\text{/1000})\text{/Nt}$$

where ER denotes the rate of ammonia excretion (mg NH_4_-N h^-1^ g^-1^), *C*_e_ is the ammonia concentration (mg L^-1^) in the experimental sample, *C*_c_ is the ammonia concentration (mg L^-1^) in the control, *V* is the seawater volume (ml) in the tank, *N* is the mussel number in the tank and *t* is the exposure time (*h*). ER values were changed to J h^-1^using the conversion value of 1mg NH_4_-N = 25 J (Elliott and Davison, 1975). The ratio of oxygen consumption to ammonia excretion (O:N) was calculated to reflect the proportion of protein relative to carbohydrates and lipids metabolized under different conditions (Widdows, 1985).

### Scope for Growth

Scope for growth was computed by the energy balance equation given by Smaal and Widdows (1994):

SFG=Ab−(R+U)

Where SFG denotes SFG (J h^-1^g^-1^), Ab represents the total absorbed energy (J h^-1^ g^-1^), *R* is the energy lost for respiration (J h^-1^g^-1^), and *U* is the energy lost for ammonia excretion (J h^-1^g^-1^).

Absorption rate (Ab) = IR (J h^-1^) × AE (%).

### Statistical Analyses

The normality and homogeneity of the data were checked by Shapiro-Wilk’s test and Levene’s test (SPSS 19.0), respectively. The effects of pH, nano-ZnO and their interactions were analyzed by two-way analysis of variance (ANOVA). If there is an interaction, the significant effects of nano-ZnO were analyzed by one-way ANOVA at each fixed pH followed by Tukey’s HSD test. Student’s t-test was applied to examine the significant effects of pH at each nano-ZnO concentration. Principal component analysis (PCA) was conducted by XLSTAT^®^2014. The measured parameters and the observations were listed in a biplot. The significant difference level was considered as *P* < 0.05.

## Results

### Seawater Chemistry

During the experiment, the salinity remained at 25.1 ± 0.3 psu, the water temperature remained at 20.1 ± 0.3°C, the normal pH was kept at 8.10 ± 0.02, and the low pH was kept at 7.69 ± 0.02 (**Table 1**). Moreover, **Table 1** also summarized the carbonate chemical parameters of seawater for all treatments. All mussels were alive during the experimental period.

**Table 1:** Seawater chemistry monitoring during the experiment (mean ± SD, *n* = 4).

Treatments	Salinity	T (°C)	pH_NBS_	TA	DIC	p CO_2_	Ωcal	Ωara
pH^∗^ZnO	(psu)		±	(μmol Kg^-1^)	(μmol Kg^-1^)	(μatm)	±	
8.1^∗^0	25.1 ± 0.2	20.0 ± 0.1	8.11 ± 0.01	2268 ± 77	2047 ± 67	365 ± 11	4.35 ± 0.24	2.74 ± 0.15
8.1^∗^2.5	25.2 ± 0.1	20.1 ± 0.3	8.10 ± 0.02	2308 ± 42	2085 ± 37	375 ± 17	4.41 ± 0.17	2.78 ± 0.11
8.1^∗^10	25.1 ± 0.2	20.1 ± 0.2	8.10 ± 0.01	2356 ± 34	2134 ± 31	391 ± 5	4.44 ± 0.09	2.80 ± 0.05
7.7^∗^0	25.2 ± 0.1	20.0 ± 0.1	7.70 ± 0.01	2331 ± 52	2261 ± 53	1062 ± 45	1.98 ± 0.05	1.25 ± 0.03
7.7^∗^2.5	25.1 ± 0.3	20.1 ± 0.2	7.69 ± 0.02	2342 ± 22	2276 ± 23	1096 ± 47	1.95 ± 0.06	1.23 ± 0.04
7.7^∗^10	25.0 ± 0.3	20.1 ± 0.2	7.71 ± 0.01	2354 ± 6	2283 ± 7	1062 ± 25	2.02 ± 0.06	1.27 ± 0.03

*pH was monitored by the pH/CO_2_ system continuously during the experiment. Salinity, temperature and total alkalinity (TA) were determined at each sampling time. Partial pressure of CO_2_ (pCO_2_), dissolved inorganic carbon (DIC), saturation degrees for calcite (Ωcal) and aragonite (Ωara) were calculated based on the above parameters.*

### Nano-ZnO Characterization

The nano-ZnO morphology was observed by both TEM and SEM. The particle diameter of ca. 15–25 nm and spheroid irregular shapes of nano-ZnO were found by TEM and SEM, respectively (Supplementary Figures S1A,B). From the X-ray diffraction patterns (Supplementary Figure S1C), all peaks of the nano-ZnO were well indexed to the hexagonal wurtzite structure of ZnO (JCPDS Card no. 01-089-0510), and there were no other impurity diffraction peaks in the spectrum, so the nanomaterials were pure nano-ZnO. DLS results showed that the particle size of nano-ZnO was affected by pH (**Table 2**).

**Table 2:** The parameters of Nano-ZnO sedimentation experiments.

Nano-ZnO (mg L^-1^)	pH	Hydrodynamic diameter (nm)	Particle dispersion index	Zeta potential (mV)
2.5	8.1	749 ± 53	0.22 ± 0.03	-15.03 ± 4.45
10	8.1	1235 ± 128	0.08 ± 0.01	-21.39 ± 5.56
2.5	7.7	1083 ± 96	0.14 ± 0.02	-15.86 ± 3.25
10	7.7	1761 ± 134	0.06 ± 0.02	-20.16 ± 4.12

### Physiological Parameters

**FIGURE 1:**
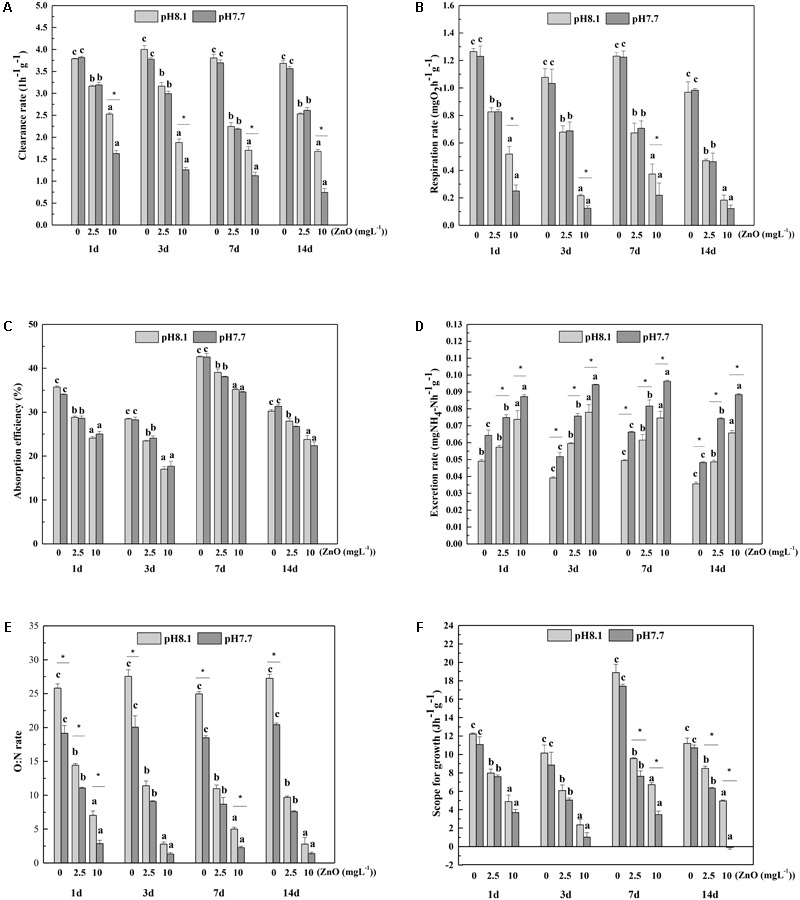
**(A)** Clearance rate (CR), **(B)** absorption efficiency (AE), **(C)** respiration rate (RR), **(D)** ammonia excretion rate (ER), **(E)** O:N ratio and **(F)** Scope for growth (SFG) of *M. coruscus* exposed to different combinations of pH and nano-ZnO for 14 days. The means denoted by different superscripts at each fixed pH are significantly different among three nano-ZnO concentrations at each sampling time (*P* < 0.05). The means sharing the asterisk between two pH levels at each fixed nano-ZnO are significantly different at each sampling time (*P* < 0.05).

CR varied from 1.1 to 4.0 L h^-1^ g^-1^ after the mussels were exposed to different treatments of pH and nano-ZnO for 14 days. High concentration of nano-ZnO resulted in a significant reduction of CR throughout the whole experiment. Low pH significantly reduced the CR of the thick shell mussels (*p* < 0.05) when the nano-ZnO was 10 mgL^-1^. CR was the lowest when the concentration of nano-ZnO was 10 mg L^-1^ under pH 7.7 (**Figure 1A**).

The AE was low, ranging from 15 to 45%. High concentration of nano-ZnO significantly reduced the AE, but there was no significant difference between the two pH levels. During the 14-day experiment, AE was reduced to the lowest value when the concentration of nano-ZnO was 10 mg L^-1^ (**Figure 1B**).

During the experiment, RR was significantly affected by the interaction of nano-ZnO and pH (**Table 3** and **Figure 1C**). Similar to CR, high nano-ZnO significantly reduced the RR of the mussels (*p* < 0.05). RR was significant reduced (*p* < 0.05) by low pH when the nano-ZnO is 10 mgL^-1^ except at day 14. The lowest value of measured RR at each time point was at pH 7.7 and nano-ZnO 10 mg L^-1^ (**Figure 1C**).

**Table 3:** Summary of two-way ANOVA results on effects of pH and nano-ZnO on clearance rate (CR), absorption efficiency (AE), respiration rate (RR), excretion rate (ER), O:N ratio, and scope for growth (SFG).

Source		CR			AE			RR		
		ZnO	pH	ZnO^∗^pH	ZnO	pH	ZnO^∗^pH	ZnO	pH	ZnO^∗^pH
	df	2	1	2	2	1	2	2	1	2
1 day	MS	4.580	0.350	0.430	161.977	0.465	2.605	0.651	0.665	0.019
	F	283.940	216.717	265.617	187.311	5.382	30.174	567.484	614.069	17.278
	P	<0.001	0.658	0.317	<0.001	0.039	12.124	<0.001	0.064	0.031
3 days	MS	4.974	0.095	0.360	302.314	3.166	63.926	0.924	0.182	0.013
	F	53.489	58.474	21.031	680.977	7.131	13.320	916.872	180.840	13.320
	P	0.084	0.084	0.165	0.481	0.774	1.226	<0.001	0.014	0.032
7 days	MS	8.233	0.052	0.338	9.710	0.778	0.140	0.609	0.184	0.001
	F	239.069	85.977	12.746	79.135	83.399	5.044	33.645	472.892	2.911
	P	0.112	0.05	2.221	0.114	0.016	0.921	0.445	0.784	0.044
14 days	MS	5.507	<0.001	0.019	89.258	1.194	2.987	0.785	0.081	0.006
	F	189.218	11.793	66.094	23.246	361.646	407.953	36.955	93.456	16.452
	P	1.245	5.681	5.121	0.488	0.987	0.557	0.663	0.412	0.018

**Source**		**ER**			**SFG**			**O:N**		
		**ZnO**	**pH**	**ZnO^∗^pH**	**ZnO**	**pH**	**ZnO^∗^pH**	**ZnO**	**pH**	**ZnO^∗^pH**
	**df**	**2**	**1**	**2**	**2**	**1**	**2**	**2**	**1**	**2**

1 day	MS	0.001	0.001	0.456	81.270	3.775	0.297	463.162	100.716	4.482
	F	91.920	113.792	0.705	324.647	15.080	1.185	885.728	192.604	8.571
	P	<0.001	<0.001	0.514	<0.001	0.002	0.339	<0.001	<0.001	0.005
3 days	MS	0.001	0.002	<0.001	91.469	6.715	0.047	723.992	63.646	16.004
	F	487.209	197.843	1.284	162.743	11.947	0.084	370.508	32.571	8.190
	P	<0.001	<0.001	0.312	0.005	<0.001	0.920	<0.001	<0.001	0.006
7 days	MS	0.001	0.002	<0.001	273.568	21.985	1.263	506.541	66.142	7.943
	F	169.565	256.020	1.392	285.058	229.201	13.171	893.046	247.185	29.684
	P	<0.001	<0.001	0.286	<0.001	<0.001	0.061	<0.001	<0.001	<0.001
14 days	MS	0.002	0.002	<0.001	110.021	29.480	8.092	746.436	53.524	13.136
	F	221.616	224.575	83.068	370.898	883.702	462.456	941.324	674.916	165.644
	P	<0.001	<0.001	<0.001	<0.001	<0.001	0.315	<0.001	<0.001	<0.001

*pH: 7.7and 8.1; Nano-ZnO: 0, 2.5 mg L^-1^ and 10 mg L^-1^.*

During the whole experiment, low pH significantly increased ER (*p* < 0.05), and ER was significantly increased with the increment of nano-ZnO concentrations except at day 1. Moreover, the highest values were observed when the mussels were subject to low pH and high nano-ZnO, and interactive effects of nano-ZnO and pH were found at day 14 (**Table 3** and **Figure 1D**).

Nano-ZnO significantly reduced O: N ratio throughout the experiment, and low pH significantly decreased the O: N ratio compared to normal pH 8.1 when nano-ZnO was absent (*p* < 0.05), and some interactive effects were also observed (**Table 3**). The effects of both pH and nano-ZnO showed similar trends and resulted in a significant low value at low pH and high nano-ZnO (**Figure 1E**).

The SFG was significantly decreased (*p* < 0.05) under high nano-ZnO condition throughout the whole experiment. Moreover, the SFG displayed positive values for all treatments except for nano-ZnO 10 mg L^-1^ and pH 7.7 at day 14. Low pH significantly decreased the SFG (*p* < 0.05) when nano-ZnO was 2.5 and 10 mg L^-1^ at days 7 and 10 (**Figure 1F**), and the lowest SFG was observed under the combination of high nano-ZnO and low pH.

**FIGURE 2:**
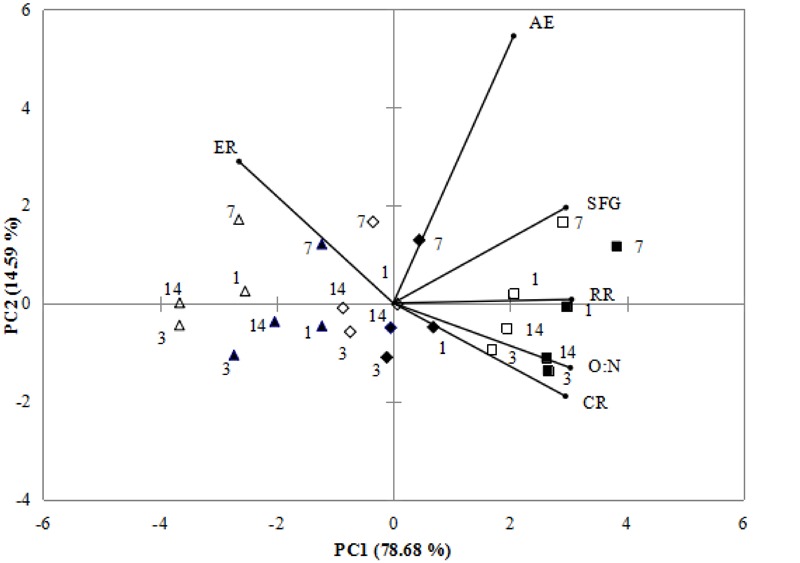
Biplot originating from principal component analysis integrating all measured variables (CR, AE, RR, ER, O:N, SFG) for four sampling times (days: 1, 3, 7, and 14) at six different treatments (■-0 mgL^-1^ × pH 8.1, □–0mgL^-1^ × pH 7.7, ♦-2.5 mgL^-1^ × pH 8.1, ♢-2.5 mgL^-1^ × pH 7.7 ▲-10 mgL^-1^ × pH 8.1, Δ-10 mgL^-1^ × pH 7.7). Both the loadings of the variables (•) and the scores of the experimental conditions were shown.

According to PCA, 93.27% of total variance was represented by the two principal components (**Figure 2**). PC1 accounted for 78.68% of total variance, the distinct response was the separation between zero concentration and high concentration of nano-ZnO, where the high levels of most physiological activities were observed, especially high levels of SFG associated with CR, O:N and RR under normal pH and 0 nano-ZnO conditions. PC2 representing 14.59% of total variance, differentiated normal pH from low pH exposed mussels, showing that high ER was correlated to low pH treatments.

## Discussion

It was reported that pH has a significant effect on the scattering behavior of ZnO nanoparticles (Bian et al., 2011), and the physical and chemical properties of nanoparticles can be affected by low pH value, thus promoting the polymerization behavior (Huang et al., 2016). The DLS results showed that nano-ZnO can agglomerate to form larger particles at low pH compared to pH 8.1. In terms of the feeding characteristics of mussels, they can ingest more nano-ZnO in the form of aggregation during acidification conditions, resulting in more accumulation of nano-ZnO in the body and leading greater biological toxicity.

From the experimental results, the combination of seawater acidification and nano-ZnO had a direct impact on the physiology of *M. coruscus*, and some interactive effects were observed. It was found that 14-d nano-ZnO exposure led to significant low CR, RR, AE, SFG, and O: N ratio, but high ER in the thick shell mussel, while the low pH had less effect on the mussels compared to high nano-ZnO. According to the existing research reports, the effect of decreased pH on CR of bivalves is different. Sui et al. (2016) found that the CR of *M. coruscus* was significantly reduced at low pH. Liu and He (2012) found that low pH resulted in decreased clearance of *Chlamys nobilis* and *Perna viridis.* However, low pH made the CR of the pearl oyster *Pinctada martensi* increased while the filtration activities of the mussel *M. galloprovincialis* were not affected by the pH reduction (Fernández-Reiriz et al., 2012). For NPs effects, nano-TiO_2_ exposure reduced CR of *M. coruscus* (Hu et al., 2017). CR of *M. galloprovincialis* increased with nano-CeO_2_ concentration but decreased over time in groups exposed to nano-CeO_2_ (Conway et al., 2014). In addition, Doyle et al. (2016) found that the adsorption of nano-TiO_2_ on the gill surface in mussels and oysters resulted in sub-lethal effects, causing gill proliferation and edema and thus affected the respiratory function, damaged mussel filtration function and food intake. In this experiment, it was speculated that nano-ZnO damaged the function of gills of the mussels, thus reducing the CR.

The results showed that the AE of *M. coruscus* was not affected by low pH. Navarro et al. (2013) found that low pH significantly reduced the AE of *M. chilensis*. However, at low pH conditions, *M. galloprovincialis* can show high AE values (Fernández-Reiriz et al., 2012). It is speculated that the low pH may increase the activity of certain digestive enzymes of *M. galloprovincialis* to promote the nutrient uptake (Areekijseree et al., 2004). From the above results, it can be seen that the effect of acidification on the digestion and absorption is different among species. NPs can enter digestive system and induce oxidative stress and lysosomal membrane changes in digestive gland of mussels (Canesi et al., 2012; Hull et al., 2013). Saggese et al. (2016) found a decreasing AE trend across the silver nanoparticles (AgNPs) concentration gradient in the mussel *Brachidontes pharaonis*. In the present study, high nano-ZnO significantly reduced AE of the mussels, which may be due to the accumulation of nano-ZnO in the digestive tube of mussels, causing severe stress responses and damage to mussel health. It can be inferred that nano-ZnO has a toxic effect on the digestive physiology of the thick shell mussel.

pH significantly reduced the RR of the mussels when nano-ZnO was 10 mgL^-1^, indicating the negative effect of acidification on mussels was enhanced under high nano-ZnO. Michaelidis et al. (2005) pointed out that seawater acidification significantly decreased the RR of the mussel *M. galloprovincialis*. Liu and He (2012) found that the RR of the noble scallop *Chlamys nobilis* was significantly reduced at pH 7.4. In contrast, researchers have pointed out that some species have a certain adaptability to seawater acidification, thus seawater acidification sometimes increases the metabolic efficiency in some species, such as the mussel *M. galloprovincialis* and the scallop *Pecten maximus* (Fernández-Reiriz et al., 2012; Sanders et al., 2013). Throughout the whole experiment, nano-ZnO always negatively affected the RR of the mussels. Due to the toxic effects of nano-ZnO on the gills, the respiration function may be impaired, and ultimately the RR was decreased. However, Hanna et al. (2013) found that after 12 weeks of exposure to nano-ZnO, RR of mussels increased with ZnO concentration, indicating that mussels may adapt nano-ZnO conditions if they were exposed for long term. Muller et al. (2014) confirmed that nano-ZnO accumulated in tissues could impair the RR of *M. galloprovincialis*.

The ER can be used as an ideal indicator of stress in mussels (Fernández-Reiriz et al., 2011). It is presumed that the stress response of the mussels to acidification leads to an increase in ER. In this study, high nano-ZnO and low pH increased the ER in *M. coruscus.* There were similar studies made by Thomsen and Melzner (2010) who pointed out that with the increase in pCO_2_, the ER of *M. edulis* was increased, while Michaelidis et al. (2005) found the ER of *M. galloprovincialis* was significantly reduced under acidic conditions. If the oyster *Pinctada mazatlanica* was exposed to high temperature, the majority of feces is amino acid catabolism products (Saucedo et al., 2004), indicating an amino acid catabolism under stress conditions. It was found that higher ER values under low pH were observed when NPs were present (Hu et al., 2017). In this study, the increase in ER may indicate a sharp increase in amino acid catabolism of the mussels exposed to high nano-ZnO and low pH. Therefore, it can be concluded that high nano-ZnO and low pH can affect the physiological metabolism of *M. coruscus*.

It is known that normal O:N for molluscs usually is higher than 30 (Fernández-Reiriz et al., 2011). When the amino acid metabolism increases (lack of food or environmental stress) the O:N values may be less than 30 (Fernández-Reiriz et al., 2011). During the whole experiment, O:N ratio was in the range of 4–28, and it was found that the O:N ratio of *M. coruscus* was negatively affected by high nano-ZnO and low pH. It is therefore speculated that high nano-ZnO and low pH can increase the protein metabolic rate of mussels.

Scope for growth is an important indicator for the impact of environmental stress on mussel physiology (Navarro et al., 2013). In the present study, when *M. coruscus* was exposed to high nano-ZnO, the SFG value became negative on day 14, which may be due to a significant decrease in CR, since most of the other parameters measured can be affected by the reduced filtration activity of mussels and the time spent on eating or breathing. In addition, high concentration of nano-ZnO significantly reduced the SFG, showing that nano-ZnO obviously damage the growth of *M. coruscus.* There was a similar study made by Hu et al. (2017) who pointed out that the NPs could significantly reduce SFG of *M. coruscus.* Acidification also resulted in a decreased SFG of the thick shell mussel at days 7 and 14 when nano-ZnO was 2.5 and 10 mg L^-1^. Similarly some studies found that long-term acidification exposure significantly reduced the growth of *M. galloprovincialis* and *Crassostrea virginica* (Michaelidis et al., 2005; Berge et al., 2006; Beniash et al., 2010). Navarro et al. (2013) pointed out that high levels of pCO_2_ in seawater had a negative impact on the health of *M. galloprovincialis*. Duarte et al. (2014) showed that elevated carbon dioxide concentrations had a negative effect on calcium deposition and the weight of *M. chilensis*. However, Fernández-Reiriz et al. (2011) found acidification caused elevated SFG of *M. galloprovincialis*, indicating the tolerance to acidification is species specific and even different within species.

The PCA separated non-nano-ZnO treatments from exposed treatments since non- nano-ZnO treatments were at positive side whereas exposed treatments were at negative side by PC1. Under non- nano-ZnO treatments, there are higher values of AE, SFG, RR, O:N, and CR. PC2 reflected pH change of the experiment, as high values of ER was positive, corresponding to the low pH treatments. According to ANOVA and PCA results, the lower CR, RR, AE, O:N ratio and SFG, and higher ER were associated with nano-ZnO exposure treatments. In this study, CR, RR, SFG and O:N ration had positive correlations, if CR, RR and O:N were reduced, SFG was also reduced (**Figure 2**). In addition, higher ER under high nano-ZnO and low pH indicated the low absorption rate and high protein catabolism, which was harmful to the growth of mussels.

## Conclusion

The impact of high nano-ZnO exposure was greater than that of low pH, the physiological parameters of mussels were affected more severely by the combined stress of seawater acidification and high concentration of nano-ZnO, i.e., the low pH enhanced the toxicity of nano-ZnO to the *M. coruscus*. The results of this study provided new insights for future understanding of the effect of nanomaterials and ocean acidification on marine organisms.

## Author Contributions

WH and YW designed and led the study. MH, LL, YS, HK, and YL performed the experiments. YS, ZL, FW, and XH analyzed data. YS and YW wrote the manuscript. All authors reviewed the manuscript.

## Conflict of Interest Statement

The authors declare that the research was conducted in the absence of any commercial or financial relationships that could be construed as a potential conflict of interest.
