# Supplementary material for: Synergistic Effects of Nano-ZnO and Low pH of Sea Water on the Physiological Energetics of the Thick Shell Mussel Mytilus coruscus
Source: Front Physiol. 2018 Jun 19;9:757. doi: 10.3389/fphys.2018.00757 (PMC6018417; doi:10.3389/fphys.2018.00757)
Supplement: Supplementary file 1 [file Image_1.PDF]

Supplementary Materials

Supplementary Fig.1 Characterization of nano-ZnO. A, SEM image; B, TEM image; C, X-ray diffractograms pattern.

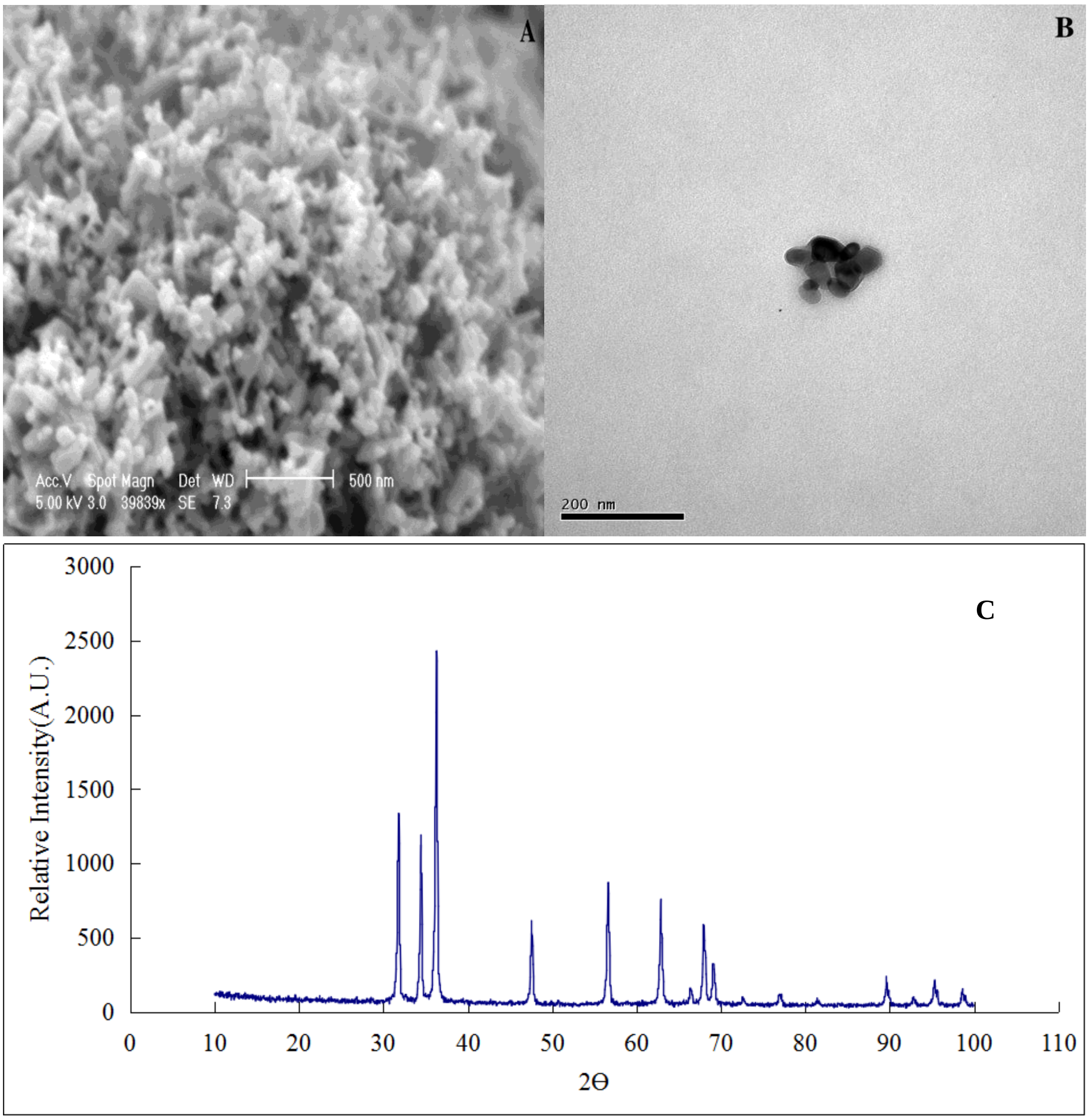

Supplementary Fig.1
